# Interest in and Preference for Long-Acting Injectable Pre-exposure Prophylaxis Among Men Who Have Sex With Men, Trans* Individuals, and Cisgender Heterosexual Women: A Global Systematic Review and Meta-Analysis

**DOI:** 10.1097/QAI.0000000000003754

**Published:** 2025-08-27

**Authors:** Haoyi Wang, Johann Kolstee, Marco Gaetani, Liana Aphami, Alejandro Adriaque Lozano, Hanne M. L. Zimmermann, Kai J. Jonas

**Affiliations:** a Department of Work and Social Psychology, Maastricht University, Maastricht, the Netherlands; and; b Viroscience Department, Erasmus Medical Centre, Rotterdam, the Netherlands.

**Keywords:** LAI-PrEP, MSM, trans* individuals, heterosexual women, interest, preference

## Abstract

**Background:**

Long-acting injectable pre-exposure prophylaxis (LAI-PrEP) offers potentials for expanding PrEP coverage and improving public health outcomes. This study synthesizes evidence on the prevalence and determinants of interest in and preference for LAI-PrEP among men who have sex with men (MSM), trans* individuals, and heterosexual women.

**Methods:**

We conducted this systematic review and meta-analysis building on quantitative studies from a previous review and new studies published in PubMed, Web of Science, and Embase up to December 31, 2023. We used a random-effects meta-analysis to pool the prevalence of LAI-PrEP interest and preference, and narratively synthesized their determinants across all key populations.

**Findings:**

We included 41 articles. Seventy-four percentage (95% CI: 71 to 78) of MSM showed interest in using LAI-PrEP, and 37% (95% CI: 29 to 44) of them preferred LAI-PrEP over other HIV prevention methods. The prevalence of interest and preference were even higher among current oral PrEP users [77% (95% CI: 70 to 84) and 43% (95% CI: 28 to 58), respectively]. Interest in LAI-PrEP among trans* individuals was similarly high at 72% (95% CI: 67 to 78), with 1 study reporting that 57% of transgender women preferred LAI-PrEP. Cisgender heterosexual women also showed a high preference for LAI-PrEP (55%, 95% CI: 40 to 70). No significant differences were found between low-/middle-income countries and high-income countries across any key populations. Overall, people with more resources and who are already aware of and using oral PrEP are likely more interested in LAI-PrEP when it becomes available.

**Conclusions:**

Continued research is essential to effectively deploy LAI-PrEP and address HIV prevention gaps. Because more countries adopt LAI-PrEP, understanding its impact and reaching underserved populations will be critical to maximizing public health benefits.

## Abstract 2

Supplemental Digital Content is Available in the Text.

## INTRODUCTION

The availability of advanced HIV biomedical prevention tools is significantly accelerating efforts to end the HIV epidemic.^1^ These tools are particularly crucial for key populations with an elevated risk of HIV acquisition,^1,2^ such as men who have sex with men (MSM), trans* individuals, and cisgender heterosexual women.

Daily and on-demand oral PrEP has proven to be highly effective in preventing HIV, and is widely accepted among key populations.^3,4^ By 2023, approximately 3.5 million individuals globally were benefiting from oral PrEP.^1^ However, its uptake varies significantly across regions, with most users in eastern and southern Africa and the United States, while other areas (eg, eastern Asia and Europe) are gradually increasing their adoption.^5–7^ Yet, the uptake is still far from reaching the United Nations 2025 targets of PrEP globally.^1,8^ In addition, the real-world effectiveness of PrEP is closely associated with its adherence,^9^ which remains a challenge globally.^10,11^ Furthermore, suboptimal adherence not only reduces the effectiveness of PrEP on the individual level, but also limits its potential public health impact,^12,13^ creating a significant barrier to HIV prevention efforts.^14^

Novel PrEP modalities, particularly long-acting injectable PrEP (LAI-PrEP), have been developed to alleviate adherence issues and the burden of daily pill taking. LAI-PrEP has demonstrated superiority over oral PrEP regimens in preventing HIV infection among all key populations.^15,16^ One such LAI-PrEP option, long-acting injectable cabotegravir, was approved for use as HIV PrEP in the United States and Europe.^17,18^ In addition, other LAI-PrEP modalities, such as lenacapavir, are under investigation and have demonstrated high efficacy.^19^ Therefore, LAI-PrEP is expected to enhance the public health impact of PrEP among those current users with suboptimal use patterns, and increasing population coverage by attracting individuals who were PrEP-naive or those who previously discontinued using oral PrEP because of adherence challenges or pill burdens.^20,21^

It is, therefore, crucial to understand the interest in and preference for LAI-PrEP regimens over other HIV prevention methods among key populations to explore their potential for broader global implementation and higher PrEP coverage. A previous systematic review reported a generally high level of interest and preference for LAI-PrEP within these populations.^22^ The review also highlighted significant variations both within and across these key groups. Despite this promising interest, the current evidence remains largely qualitative, only included studies up to September 2021 when there was no authorized LAI-PrEP regimen globally, and mostly based on studies conducted in a US context. Given the increasing interest in long-acting modularities in HIV prevention globally,^23^ there is a clear need for further meta-analytical studies that aim more globally to provide up-to-date robust evidence with broader geographic representation. More importantly, such meta-analyses should particularly examine regional patterns—including analyses stratified by World Bank income levels—to identify potential regional and structural differences in LAI-PrEP interest and preference across key populations. These insights could inform tailored implementation strategies to optimize LAI-PrEP rollout across diverse settings.

To inform and support future LAI-PrEP research and implementation efforts, it is essential to identify the facilitators and barriers influencing interest and preference for LAI-PrEP. In addition, it is important to explore whether these determinants differ from those driving interest in and uptake of oral PrEP, as summarized in previous systematic reviews that focused on oral PrEP.^24,25^ A deeper understanding of these variations and heterogeneity, particularly those shown to be statistically significant on a global scale, will enable future research to build more effectively on existing findings, leading to more efficient studies and more precise public health strategies. At this early stage of LAI-PrEP introduction, it is also vital to identify gaps in the current literature, such as underexplored or inconsistently assessed factors across key populations and regions. Mapping these gaps will offer a clearer picture of the research landscape and guide the development of a more coherent and strategic research agenda, which can be particularly helpful to identify under-researched populations and determinants. However, to date, no previous systematic reviews have provided these critical insights on LAI-PrEP.

Taken together, there is a clear need for a comprehensive and robust meta-analytical summary of current interest and preference for LAI-PrEP. In addition, systematically synthesizing the determinants of LAI-PrEP interest and preference among key populations, alongside identifying key research and evidence gaps, can be crucial. Therefore, we present an updated global systematic review and meta-analysis, aimed at providing a detailed meta-analytical summary of LAI-PrEP interests and preferences among MSM, trans* individuals, and cis-gender heterosexual women, along with their determinants on a global scale.

## METHODS

### Selection Criteria and Search Strategy

For this updated systematic review and meta-analysis, all original studies were eligible for inclusion if they were empirical studies reporting on quantitative or mixed-method analytic findings on the proportion or determinants of the interest or preference of long-acting injectable PrEP among MSM, trans* individuals, and cisgender heterosexual women. Original qualitative studies, opinion pieces, letters to the editors, and other systematic reviews/meta-analyses were not considered eligible for inclusion. To be eligible to be included in our meta-analysis, studies must report the total number of participants, the total number or proportion of participants who were interested in or preferred to use LAI-PrEP. Definitions of LAI-PrEP interest and preference were recorded as a note.

We used 2 search methods for this updated systematic review and meta-analysis. First, we built on the findings from a previously published systematic review of the values and preferences regarding the use of injectable pre-exposure prophylaxis to prevent HIV acquisition.^22^ That review systematically searched for the same end points as this updated systematic review and meta-analysis and included articles published between January 1, 2010, and September 27, 2021.^22^ We reviewed all the studies included in that systematic review (n = 62). We only included quantitative and mixed-methods studies for our determinant synthesis and meta-analysis. Second, we further sought to include articles published between September 27, 2021, and December 31, 2023, by searching PubMed, Web of Science, and Embase, using the combined terms by the population, intervention, and outcome (PIO) framework, which included 3 main constructs: MSM/women/trans*/current oral PrEP users AND injectable modalities/PrEP/prevention AND HIV.

### Data Extraction and Quality Assessment

Data extraction and quality appraisal were performed by A.A.L., L.A., and M.G., and verified by H.W. The Newcastle–Ottawa Scale for nonrandomized studies was used to assess the methodological quality of the included studies with a cohort study design.^26^

#### Meta-Analysis

We first extracted the total number of participants for all articles that contained empirical evidence on the number/proportion of the interest or preference of LAI-PrEP. We then extracted the absolute number of participants who reported interest or preference for LAI-PrEP separately by the study population. In terms of multicenter studies, which reported independent country-specific data, we extracted the data by specific countries instead of treating the multicenter data homogenously. In terms of studies, which reported independent data on specific subpopulations or intersectional populations, such as MSM and MSM oral PrEP users, we first extracted the data on the general population, and we then extracted the specific data focus on the subpopulations. In addition, to explore regional and structural differences in LAI-PrEP interest and preference across the key populations, we categorized the data by the region of origin of the included studies, classifying them as either “High-Income Countries (HICs)” or “Low- and Middle-Income Countries (LMICs)” based on income level, as defined by the World Bank.^27^ To assess differences between HICs and LMICs, we conducted subgroup meta-analyses and meta-regressions. These analyses were performed only when at least 2 studies per end point and key population were available for each region.

For all meta-analyses, we used a random-effects model and the DerSimonian–Laird method to estimate the model on the proportion. The DerSimonian–Laird *Q* test and *I*^2^ values were used to assess heterogeneity, with low, moderate, and high heterogeneity corresponding to *I*^2^ values of 25%, 50%, and 75%. In addition, heterogeneity τ was assessed in this study. Publication bias was assessed by inspecting funnel plots using a rank correction test. To compare the difference in the interest and preference for LAI-PrEP among all key populations with at least 2 included studies per region, we fit a univariable meta-regression model with region (HICs vs. LMICs) as the covariate. Statistical significance was assessed at *P* < 0.05. The statistical analysis was performed using R (version 4.5.1).

#### Determinant Systematic Synthesis

For all articles that contained empirical evidence on the determinants of LAI-PrEP interest, we first coded the articles based on the study populations. Then, we extracted all the determinants/factors the original study reported in the univariable (unadjusted) analyses for each study population. We then extracted all the determinants/factors the original study supported with statistically significant evidence based on the adjusted effect size with 95% confidence intervals or *P* < 0.05. Given the heterogeneous measurements of similar determinants/factors from different included studies, we did not further conduct a meta-analysis to pool the effect size of each determinant. Instead, we summarized all the reported investigated determinants of LAI-PrEP interest among each study population using a narrative approach. We summarized the determinants with the directions of the associations found to be statistically significant in the original studies.

## RESULTS

### Research Selection and Characteristics

**FIGURE 1.**
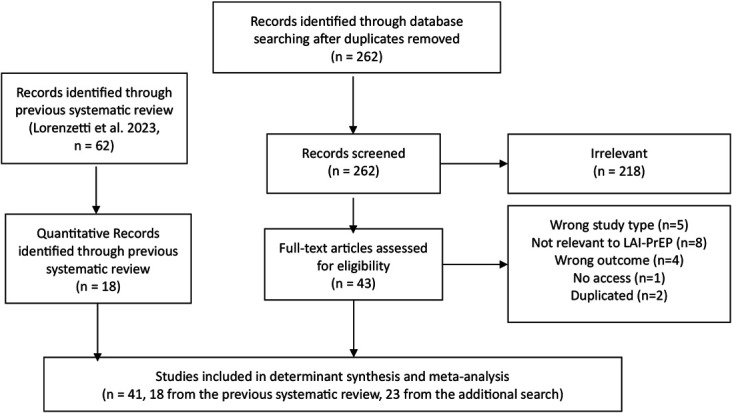
PRISMA flow diagram of the study selection process.

Of the 62 studies included in the previous systematic review,^22^ 18 quantitative studies were included in this systematic review and meta-analysis. Our additional search strategy identified 262 studies after removing duplicates. We excluded 218 studies after screening the titles and abstracts. Forty-three studies remained for full-text screening, and 20 were excluded, leaving 23 studies to be included. As a result, a total of 41 studies were included in this systematic review and meta-analysis. Figure 1 shows the selection procedure in this study.

Of the 41 studies, 12 originated from LMICs, 4 were global multicenter studies, and 25 originated from the HICs, of which 19 were from the United States alone and only 3 were from Europe. In addition, of these 41 studies, only 2 were cohort studies, 35 had a cross-sectional design, 2 had a mixed-method design, and 2 were observational studies alongside randomized control studies (see File, Supplemental Digital Content, http://links.lww.com/QAI/C568). Quality assessments were summarized in Supplemental Digital Content (see File, http://links.lww.com/QAI/C568). Overall, of the 41 included studies, 34 (83%) had good quality assessment, with 7 (17%) having fair quality assessment. There was no evidence of a publication bias in this study.

### Meta-Analysis of LAI-PrEP Interest and Preference

#### LAI-PrEP Interest and Preference Among MSM

**FIGURE 2.**
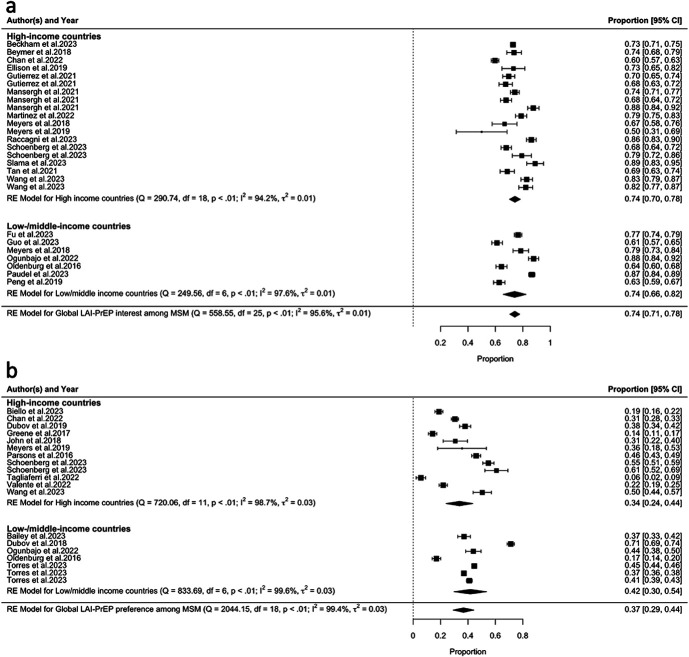
Forest plot of the prevalence of LAI-PrEP (A) interest and (B) preference among MSM.

Globally, 26 studies reported LAI-PrEP interest among MSM, of which 19 were conducted in the HICs and 7 were conducted in LMICs. Based on these studies, a pooled 74% (95% CI: 0.71 to 0.78, τ^2^ = 0.01, I^2^ = 95.60%) of MSM showed interest in using LAI-PrEP to prevent HIV globally (Fig. 2A). No significant difference in LAI-PrEP interest among MSM was found between HICs and LMICs [HICs: 74% (95% CI: 0.70 to 0.78, τ^2^ = 0.01, I^2^ = 94.20%); LMICs: 74% (95% CI: 0.66–0.78, τ^2^ = 0.01, I^2^ = 97.60%); *P* = 0.966].

In addition, 19 studies reported MSM's preference for LAI-PrEP compared with other HIV prevention methods globally, of which 12 were conducted in the HICs and 7 were conducted in LMICs. A pooled 37% (95% CI: 0.29 to 0.44, τ^2^ = 0.03, I^2^ = 99.40%) of MSM were found to prefer LAI-PrEP to prevent HIV to other HIV prevention methods globally (Fig. 2B). MSM who were living in the LMICs had higher preference for LAI-PrEP than MSM who were living in the HICs, yet this difference was not significant [HICs: 34% (95% CI: 0.24 to 0.44, τ^2^ = 0.03, I^2^ = 98.70%); LMICs: 42% (95% CI: 0.30 to 0.54, τ^2^ = 0.03, I^2^ = 99.60%); *P* = 0.314].

#### LAI-PrEP Interest and Preference Among MSM Who Were Current PrEP Users

**FIGURE 3.**
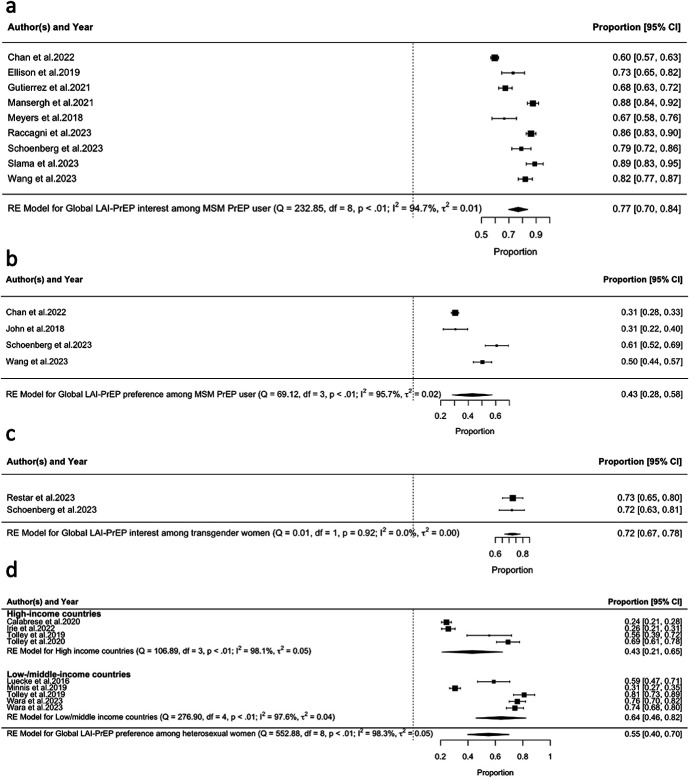
Forest plot of the prevalence of LAI-PrEP (A) interest and (B) preference among MSM current oral PrEP users, (C) LAI-PrEP interest among trans* individuals, and (D) LAI-PrEP preference among heterosexual women.

Globally, 9 studies reported LAI-PrEP interest among MSM who were current PrEP users. All of these studies were conducted in HICs. Based on these studies, a pooled 77% (95% CI: 0.70 to 0.84, τ^2^ = 0.01, I^2^ = 94.70%) of them showed interest in using LAI-PrEP to prevent HIV (Fig. 3A).

In addition, 4 studies reported the preference of MSM who were current PrEP users for LAI-PrEP compared with other HIV prevention methods. All of these studies were conducted in HICs. A pooled 43% (95% CI: 0.28 to 0.58, τ^2^ = 0.02, I^2^ = 95.70%) of MSM who were current PrEP users found to prefer LAI-PrEP to prevent HIV to other HIV prevention methods (Fig. 3B).

#### LAI-PrEP Interest and Preference Among Trans* Individuals

Globally, 2 studies reported LAI-PrEP interest among transgender women. Of these 2 studies, 1 was conducted in HICs and 1 was conducted in LMICs. Based on these 2 studies, a pooled 72% (95% CI: 0.67 to 0.78, τ^2^ = 0.00, I^2^ = 0.00%) of transgender women showed interest in using LAI-PrEP to prevent HIV (Fig. 3C). In addition, there is only 1 study that reported LAI-PrEP preference among transgender women. Based on this study, 57% of transgender women would prefer LAI-PrEP to prevent HIV to other HIV prevention methods.^28^ However, no study reported LAI-PrEP interest or preference among transgender men or other gender-diverse populations.

#### LAI-PrEP Interest and Preference Among Cisgender Heterosexual Women

Globally, 7 studies reported LAI-PrEP preference among heterosexual women, of which 3 were conducted in HICs, 4 were multicenter studies in LMICs, with 1 multicenter study also reporting data from the United States. Based on these 7 studies, a pooled 55% (95% CI: 0.40 to 0.70 τ^2^ = 0.05, I^2^ = 98.30%) of cisgender heterosexual women would prefer LAI-PrEP to prevent HIV to other HIV prevention methods (Fig. 3D). Heterosexual women who were living in the LMICs had higher preference for LAI-PrEP than heterosexual women who were living in the HICs, yet this difference was not significant [HICs: 43% (95% CI: 0.21 to 0.65, τ^2^ = 0.05, I^2^ = 98.10%); LMICs: 64% (95% CI: 0.46 to 0.82, τ^2^ = 0.04, I^2^ = 98.30%); *P* = 0.154].

#### Determinants Synthesis of LAI-PrEP Interest

Among the 41 studies included in this meta-analysis and systematic review, 36 examined determinants of interest in LAI-PrEP among MSM, cisgender heterosexual women, and trans* individuals. Most of these studies (30 of 36) focused on MSM. Across populations, several shared determinants were frequently investigated, including sociodemographic (eg, age, education, employment), psychosocial (eg, perceived HIV risk, HIV-/PrEP-related stigma), and behavioral factors (eg, number of sexual partners, condom use). In addition to these cross-cutting themes, population-specific factors were also examined—for example, contraceptive use among cisgender women and gender affirmation-related concerns among trans* individuals. Importantly, the significance and direction of associations varied by key populations, highlighting both commonalities and key differences in the factors determining LAI-PrEP interest.

#### Determinants Synthesis of LAI-PrEP Interest Among MSM

Globally, 29 studies reported empirical investigation and evidence on the determinants of LAI-PrEP interest or preference among MSM. More than 75 different sociodemographic, psychosocial, behavioral, and LAI-PrEP-related determinants were investigated in the included studies (Table 1). Of these, 45 were significantly associated with LAI-PrEP interest, with variations among studies.

**TABLE 1.** Determinants of LAI-PrEP Interest and Preference Among MSM and Current MSM Oral PrEP Users

Determinant	Study Reporting the Determinant	Study Reporting Statistically Positive Significant Association	Study Reporting Statistically Negative Significant Association
**MSM**			
Sociodemographic			
Age (ref. younger)	^28–47^	^31,37,41^	^29,36,38,46^
Circumcised (ref. no)	^39^		^39^
Did not receive health care because of cost	^45^		
Divorced/widowed (ref. no)	^36,42,43^	^42^	
Education (ref. lower)	^31–41^	^32,37^	^33,34,42,48^
Employment (ref. no)	^29,32,35,36,39,41,42,44–46,49,50^	^39^	
Ethnicity	^28,32,33,35–38,40,42–44,46–49,51,52^		
Hispanic/Latino (ref. no)		^28,32,35,40^	
White (ref. no)		^38,46^	^28,32,40^
Black (ref. no)		^32,35,40^	^38,46^
Mixed/other (ref. no)		^28,32^	^38^
Financial hardship	^29,45^		
Have primary care provider (ref. no)	^45,52^	^45^	
Housing	^35,46^		
Importance of religion	^49^		
Income (ref. low)	^28–30,32,33,35–37,41–43,45,51,53^	^37,41,53^	
Insured/insurance access (ref. no)	^28,32,35,40,44–46,48,52^	^35^	
Migrant status	^29,32,33,42,44,48^		
Muslim (ref. no)	^39,45^		^39^
Place of residence	^28–31,34,38,40,45,49,54^		
Single (ref. no)	^29,34,35,39,45,50,51^	^45^	
Socioeconomic positions	^34,48^		
Psychosocial			
Child sexual abuse (ref. no)	^44^	^44^	
Cisgender identity (ref. no)	^28,39^	^39^	^28^
Concerns about protection of LAI-PrEP (ref. Lower)	^33^		^33^
Depression (ref. lower)	^31,49^	^31^	
Fear of HIV (ref. none)	^34,48^	^34^	
Fear of needles	^33^		
Health risk/side effects	^33,41^		
HIV knowledge	^31,36,42,43^		
Interest in psychobehavioral support services (ref. no)	^48^	^48^	
Loneliness	^30^		
PEP awareness	^42^		
Perceived appropriateness of PrEP candidate	^51^		
Perceived concern about HIV (ref. no)	^32^	^32^	
Perceived risk of HIV	^32,34–36,43^		
Perceived risk of STI	^36^		
PrEP is affordable	^41^		
Providers are judgmental	^41^		
Risk compensation	^36^		
Self-perception of health	^48^		
Self-stigmatized PrEP	^28^		
Sexual orientation	^29,31,32,34–36,39,41–43,45,46,48,52^		
Sexual well-being	^30^		
Struggling with taking a daily pill (ref. no)	^41,53^	^41,53^	
Willingness to pay for PrEP (ref. unwilling)	^31^	^31^	
Willingness to take daily PrEP (ref. unwilling)	^30,40,42,51^	^30,40,42^	
Behavioral			
Age of first intercourse (ref. younger)	^36,43^		^43^
Alcohol dependency	^31,36,43,46,50,52^		
Chemsex (ref. no)	^29,30,33,34,42,47,48,51^	^42^	
CAI in the preceding 3/6 months (ref. no)	^28,30–32,34,35,38,40,44,48,52,54^	^28,40^	
CAI with partners with HIV	^32^		
Condom use frequency/consistency (ref. no)	^32,36,39,42,45,48^		
Ever got tested for HIV (ref. no)	^42^	^42^	
Ever used PEP	^30,42^		
Female partners	^33^		
Frequency of HIV testing (ref. lower)	^30–32^		
Geosocial networking app use	^45^		
Group sex	^39^		
Have partners with HIV (ref. no)	^29,32,34,35,42^		^32,34^
Have sex with female	^42^		
History of arrest	^46^		
Intimate partner violence (IPV)			
Physical IPV victimization	^43,46^		
Physical IPV perpetration	^43^		
Any physical IPV	^43^		
Sexual IPV victimization	^43,46^		
Sexual IPV perpetration	^43^		
Any sexual IPV	^43^		
Monitoring IPV victimization (ref. no)	^43,46^	^46^	
Monitoring IPV perpetration	^43^		
Any monitoring IPV (ref. no)	^43^		^43^
Controlling IPV victimization (ref. no)	^43,46^		^43^
Controlling IPV perpetration	^43^		
Any controlling IPV	^43^		
Emotional IPV victimization (ref. no)	^43,46^		^43^
Emotional IPV perpetration	^43^		
Any emotional IPV	^43^		
Any IPV victimization	^43^		
Any IPV perpetration	^43^		
Any IPV	^43^		
Knowing someone with HIV (ref. no)	^34^		^34^
Number of partners in the preceding 3/6 months (ref. less)	^30–32,35,36,39,40,42–45,47,48^	^30,36,43,48^	
Oral PrEP awareness (ref. no)	^30,34,36,37,42,45^	^30,37,42^	
Oral PrEP uptake/regimen			
Current user (ref. no)	^28–30,39–41,53^	^28,40,41^	
Daily regimen (ref. no)	^30,38,46,47^	^30,38,46^	
Early adopter (ref. no)	^29,53^	^29,53^	
Former user (ref. no)	^28–30,39,40,53^	^28,40^	^29^
Naive (ref. no)	^28–30,39,40,53^		^28–30,40^
Suboptimal oral PrEP adherence (ref. no)	^40,47^		^47^
Oral PrEP discontinuation	^29,40^		
Oral PrEP uptake frequency	^51^		
Oral PrEP use history (ref. <1 yr)	^33^		^33^
PrEP eligibility (overall risk) (ref. no)	^35,37^	^37^	
Sexual position	^34–36,39^		
Sexually active/having partners (ref no)	^31,34,44,46^	^44^	
STI diagnosis	^29,30,32,34–36,43,45,47,48^		
STI testing	^31,32,52^		
Substance use in general (ref. no)	^32,40,46,52^	^46^	
Transactional sex (ref. no)	^29,42,45^		^29^
Used any health services	^31^		
LAI-PrEP related			
Challenging/disadvantageous clinical visit intervals for injections (ref. no)	^33,41,53^		^53^
Concerns about postinjection pain	^53^		
Costs of LAI-PrEP	^41^		
Familiar/awareness of LAI-PrEP	^51^		
Injection tolerance	^53^		
**MSM current oral PrEP users**			
Sociodemographic			
Age (ref. younger)	^29,33,35,41,47^	^41^	^29^
Black or Hispanic (ref. no)	^33,35,47^	^35^	
Education (ref. lower)	^33,35,41^	^33^	
Employment	^35,41^		
Financial hardship (ref. low)	^29^	^29^	
Housing	^35^		
Income (ref. low)	^33,35,41,53^	^41,53^	
Insured/insurance access	^35^		
Migrant	^29,41^		
Relationship status	^29,35^		
Sexual orientation	^35,41^		
Psychosocial			
Fear of needles	^33^		
Health risk/side effects	^33,41^		
Perceived risk of HIV	^35^		
PrEP is affordable	^41^		
Providers are judgmental	^41^		
Taking pill daily is a burden (ref. no)	^53^	^53^	
Behavioral			
Being early adopter (ref. no)	^53^	^53^	
CAI in the preceding 3/6 mo	^35^		
Chemsex	^29,33,47^		
Have partners with HIV	^35^		
Number of partners in the preceding 3/6 mo	^35,47^		
Suboptimal oral PrEP adherence (ref. no)	^29,47^	^47^	
Oral PrEP regimen	^47^		
Oral PrEP use history (ref. <1 yr)	^33^		^33^
PrEP eligibility (overall risk)	^35^		
Sexual position	^35^		
STI diagnosis	^29,35,47^		
Transactional sex	^29^		
LAI-PrEP related			
Challenging/disadvantageous clinical visit intervals for injections (ref. no)	^41,53^		^53^
Concerns about postinjection pain (ref. no)	^53^		
Concerns about protection evidence (ref. lower)	^33^		^33^
Costs of LAI-PrEP	^41^		
Injection intervals (ref. disadvantage)	^33,53^		^53^
Injection tolerance	^53^		

Among the sociodemographic determinants found to be statistically significant, MSM who were employed, had a primary care provider, higher income, insurance access, or were single, were more likely to express interest in LAI-PrEP. Conversely, MSM who were circumcised or of Muslim faith were less likely to show interest in LAI-PrEP. Evidence was inconsistent regarding the interest in LAI-PrEP among MSM based on their age, education, and ethnicity.

Regarding psychosocial determinants found to be statistically significant, MSM who experienced childhood sexual abuse, had depression, reported higher levels of fear of HIV, showed interest in psychobehavioral support services, had higher perceived concern about HIV, struggled with daily pill taking, were willing to pay for PrEP, and those willing to take daily PrEP, showed higher interest in LAI-PrEP. In contrast, MSM with high concerns about the effectiveness of LAI-PrEP were less likely to be interested.

For behavioral determinants found to be statistically significant, MSM who had engaged in chemsex, condomless anal intercourse (CAI), had ever tested for HIV, experienced monitoring intimate partner violence, had a higher number of sexual partners, were aware of oral PrEP, were current oral PrEP users, early adopters of oral PrEP, were eligible for oral PrEP, sexually active, or used substances, were shown to have a higher interest in LAI-PrEP. Conversely, MSM who were older at first intercourse, used condoms, had partners living with HIV, experienced controlling and emotional intimate partner violence, knew someone with HIV, were oral PrEP naive, had optimal adherence to oral PrEP, had a longer history with using oral PrEP, or had engaged in transactional sex, were shown to have lower interest in LAI-PrEP. Evidence regarding former oral PrEP users' interest in LAI-PrEP was inconsistent.

Regarding LAI-PrEP-related determinants, MSM who found the clinical intervals of LAI-PrEP challenging were significantly less likely to be interested in LAI-PrEP.

#### Determinants of LAI-PrEP Interest Among MSM Who are Current Oral PrEP Users

Six studies reported empirical investigation and evidence on the determinants of LAI-PrEP interest among MSM who are current PrEP users. Thirty-five different determinants were investigated on sociodemographic, psychosocial, behavioral, and LAI-PrEP-related levels, among which 12 were statistically significant (Table 1).

Among the sociodemographic determinants found to be statistically significant, MSM who are current oral PrEP users who identified as non-Black or Hispanic, had higher education, and were not experiencing financial hardship were more likely to express interest in LAI-PrEP. However, there was no consistent evidence regarding the association between age and interest in LAI-PrEP. Regarding psychosocial determinants, MSM who are current oral PrEP users who considered daily pill taking a burden were more likely to express interest in LAI-PrEP. For behavioral determinants, MSM who are current oral PrEP users who were early adopters of oral PrEP and those with suboptimal adherence to oral PrEP were more likely to express interest in LAI-PrEP, whereas MSM who are current oral PrEP users with a longer history of oral PrEP use were less likely to be interested in LAI-PrEP. Concerning LAI-PrEP-related determinants, MSM who are current oral PrEP users who found the clinical intervals of LAI-PrEP challenging, had concerns about its effectiveness, or were uncomfortable with the injection intervals were significantly less likely to be interested in LAI-PrEP.

#### Determinants of LAI-PrEP Interest Among Cisgender Heterosexual Women

Globally, 5 studies reported empirical investigation and evidence on the determinants of LAI-PrEP interest among cisgender heterosexual women (Table 2). Twenty-nine different determinants were investigated on sociodemographic, psychosocial, behavioral, and LAI-PrEP-related levels, with only 8 determinants reported with statistically significant evidence (Table 2).

**TABLE 2.** Determinants of LAI-PrEP Interest Among Cisgender Heterosexual Women

Determinant	Study Reported	Study Reporting Positive Statistically Significant Association	Study Reporting Negative Statistically Significant Association
Sociodemographic			
Age	^55–57^		
Did not receive health care because of cost (ref. no)	^56^	^56^	
Education (ref. lower)	^55,57^		^55^
Employment	^56,57^		
Income	^56^		
Insurance status	^56^		
Marital status	^55^		
Place of residence	^55,57,58^		
United States (ref. no)			^58^
South Africa (ref. no)		^55^	
Zimbabwe (ref. no)		^55^	
Relationship status	^56^		
Socioeconomic positions	^55^		
Psychosocial			
Altruism	^57^		
Comfort discussing PrEP with provider	^56^		
Cost to travel to clinic	^58^		
Interest in community PrEP delivery	^58^		
Perceived concern about HIV	^56,58^		
Perceived risk of HIV	^57^		
Personal benefits	^58^		
Physical experience	^58^		
PrEP stigma (ref. no)	^56,57^	^56^	
Behavioral			
Alcohol dependency	^57^		
Condom use (ref. no)	^56–58^	^56^	
Contraception use (ref. no)	^57–59^	^57,59^	
HIV testing	^56^		
Number of sexual partners (ref: 0 partner)	^56,57^	^56^	
Oral PrEP history/status	^57^		
STI diagnosis	^56^		
Transactional sex	^56^		
Time travel to clinic	^57^		
Visited health care provider	^56^		
LAI-PrEP related			
Product attributes (ref. low)	^58^	^58^	

Among the statistically significant sociodemographic determinants, heterosexual women who received health care because of cost and who live outside the middle- and low-income countries such as South Africa or Zimbabwe were more likely to express interest in LAI-PrEP. Conversely, those with higher education and living in the high-income countries such as the United States were less likely to show interest. Regarding psychosocial determinants, only cisgender heterosexual women who experienced PrEP stigma were more likely to have a higher LAI-PrEP interest. For behavioral determinants, cisgender heterosexual women who reported consistent condom use, those who reported using contraception, and those who had or had a higher number of sexual partners were more likely to express interest in LAI-PrEP. Concerning LAI-PrEP-related determinants, only cisgender heterosexual women who had higher attributes of LAI-PrEP were likely to express interest in LAI-PrEP.

#### Determinants of LAI-PrEP Interest Among Trans* Individuals

Globally, there was only 1 study reporting empirical investigation of and evidence on the determinants of LAI-PrEP interest among trans* individuals.^60^ The statistically significant determinants identified included discussing HIV services with a health care provider and discussing HIV services with sexual partners.

## DISCUSSION

Overall, this analysis, which represents the largest quantitative meta-analytical synthesis on LAI-PrEP interest and preference among key populations to date, found that most MSM, trans* individuals, and cisgender heterosexual women included in the reviewed studies expressed interest in using LAI-PrEP across the globe. Notably, no significant regional differences were observed between high-income countries (HICs) and low- and middle-income countries (LMICs). In addition, we observed a significant increase in the number of studies on this topic within the past 2 years compared with the previous decade (23 vs. 18), highlighting the growing importance of generating global evidence on LAI-PrEP interest and preference to inform and enhance HIV prevention strategies worldwide.

Pooled across included studies, interest in using LAI-PrEP was highest among MSM (74%) and even higher among MSM who are current PrEP users (77%). This high interest in LAI-PrEP may reflect the higher awareness of oral PrEP observed in this group and the normalization of PrEP use during the past decade among these communities.^2,29,61^ Notably, more than a third of MSM (37%) preferred LAI-PrEP to other HIV prevention options, highlighting opportunities to engage this key population further. The higher proportion of current oral PrEP users interested in LAI-PrEP (43%) suggests that many could benefit from switching to this new modality, potentially improving adherence and offering greater convenience. Interest in LAI-PrEP among trans* people was nearly as high as that among MSM (72%), indicating a readiness in this group to adopt this new form of PrEP. The significant preference for LAI-PrEP over other HIV prevention methods among trans* people (57%) further supports expanding HIV prevention coverage in this population. However, with limited studies focusing on trans* individuals, more research is urgently warranted. Encouragingly, cis-gender heterosexual women also showed a high preference for LAI-PrEP (55%), a promising finding given that globally this group is less likely to use PrEP compared with MSM and trans* populations. LAI-PrEP could, therefore, help address the unmet demand for oral PrEP among this population.^62^

It is important to note that, despite our efforts to maintain a global focus, approximately half of the studies (19 out of 41) were conducted in the United States, with very few representing other regions, such as Europe (3 out of 41). This focus is understandable given the availability of cabotegravir, an LAI-PrEP product, in the United States since 2022, whereas it has not yet been officially introduced in many other contexts. This has provided a test case in a jurisdiction where multiple PrEP options are accessible.^17,63^ Notably, our analysis found no significant differences in LAI-PrEP interest and preference across key populations—including MSM, trans* individuals, and cisgender heterosexual women—between HICs and LMICs. This suggests that LAI-PrEP could be a widely accepted HIV prevention tool not only in HICs but also in LMICs, potentially supporting regional HIV control efforts. This prospect is further strengthened by voluntary licensing agreements signed by manufacturers of cabotegravir and lenacapavir, which aim to facilitate generic, lower-cost LAI-PrEP production for many LMICs.^64,65^ However, significant research gaps remain, particularly in LMICs across sub-Saharan Africa, Latin America, and South/Southeast Asia, as well as in other high- and middle-income regions such as Europe (where LAI-PrEP has recently gained regulatory approval^18^). Further studies are needed to assess interest, accessibility, and implementation opportunities in these settings. Addressing these gaps will be essential to fully realizing the global health potential of LAI-PrEP.

The determinants of interest in LAI-PrEP across studies were diverse but some patterns were observed. Across the studies assessed, MSM were more interested in LAI-PrEP if they came from a more privileged background (eg, employed, higher income, insured, White), perceived HIV as a threat, and believed in the efficacy of PrEP as a preventative tool. In addition, they were more interested in LAI-PrEP, unsurprisingly, if they were sexually adventurous (eg, higher number of sex partners, practice chemsex) and were already exposed to PrEP either through their own use or awareness of others' use. Very few studies investigated the determinants of LAI-PrEP interest among cisgender heterosexual women and trans* people.

The results obtained in this meta-analysis mirror those found in previous studies examining the meanings and values of LAI-PrEP before 2021.^22^ These results, based on studies globally for the past 3 years, demonstrate a growing level of interest in LAI-PrEP among key populations affected by HIV. In particular, MSM and trans* people are aware and knowledgeable about oral PrEP and are ready to pick up new PrEP modalities. LAI-PrEP provides an incredible opportunity to increase HIV prevention coverage globally, but a targeted promotion of this new modality is required to engage key populations. Understanding who is interested in LAI-PrEP is critical for health systems and the community sectors to understand, so they may most effectively inform people and help them pick up this effective HIV prevention tool. However, further detailed data are needed at the country and regional levels to better determine the geographical differences or variations in LAI-PrEP interests and preferences.

These findings have several important implications for research, policy, and practice. First, high levels of interest and preference for LAI-PrEP among key populations—particularly MSM and trans* individuals—suggest that these groups are well positioned to serve as early adopters during global rollout efforts. Policymakers and implementers should prioritize these populations in the initial phases of LAI-PrEP introduction. Second, the high proportion of individuals expressing a preference for LAI-PrEP highlights the need for further research into the underlying drivers of this preference, including convenience, stigma, and adherence challenges with oral PrEP. Importantly, similar to the early uptake patterns of oral PrEP, those with greater socioeconomic advantage or access to health services may disproportionately benefit from LAI-PrEP. Health systems must plan well to ensure that people from key communities who are less privileged are prioritized when health communication about LAI-PrEP occurs and when considering subsidization of the cost of LAI-PrEP for end users.^66^ Furthermore, these results point to the need to speak to key communities affected by HIV about the range of HIV prevention strategies at their disposal. Some may transition from condoms to oral PrEP to LAI-PrEP, while others may prefer different pathways. Clear, inclusive, and adaptive health communication will be essential to maximizing the impact of LAI-PrEP within comprehensive HIV prevention programs to improve overall HIV prevention coverage.^7^

Several limitations exist with this analysis. One key limitation is that in countries where an LAI-PrEP product is not yet available, participants in research studies must provide hypothetical responses regarding their interest and preference for a product they had not yet used. Furthermore, uncertainties around the availability and cost of the product may influence their responses. The rollout of oral PrEP has shown that uptake can be significantly affected by factors such as where the medication can be obtained, who provides it, and how much it costs.^67–69^ Therefore, studies that consider access pathways and the pricing of LAI-PrEP are needed to fully understand their impact on interest and preference. Another limitation is the variation in how LAI-PrEP interest and preference were defined across the included studies. For our meta-analysis, we only included studies that reported absolute number of participants interested in or preferring LAI-PrEP, excluding those that measured these factors differently, such as using median scores (eg, Hsu et al^30^). This selection could introduce bias into our findings. Similarly, in our synthesis of determinants, the substantial heterogeneity in how variables were measured across included studies precluded meta-analytical quantification. Nevertheless, through a narrative synthesis that documented the global research landscape, we summarized both the statistical significance and direction of associations for each examined determinant. This approach offers valuable insights for the ongoing global introduction and evaluation of LAI-PrEP by identifying characteristics that consistently predict greater interest across key populations to inform tailored implementation strategies. It also highlights determinants with inconsistent or contradictory associations across studies, which warrant further investigation to build a more robust evidence base. Therefore, despite the absence of meta-analytical evidence, we consider our findings to be valid and reliable, with continued relevance for shaping future research agendas, promoting the collection of more standardized data, and informing more efficient global health policies and actions. Finally, another limitation is the lack of differentiation in preferences for different injection intervals of LAI-PrEP, especially because new long-acting injectable modalities are emerging and proving effective.^15,19^ Because most of the included studies focused on a 2-monthly LAI-PrEP regimen, our findings may not be generalizable to newer, twice-yearly LAI-PrEP options.

## CONCLUSIONS

In conclusion, our analysis found high levels of interest in LAI-PrEP among key populations affected by HIV, though notable differences persist between groups and regions. Overall, people who have more resources and who are already aware and using oral PrEP are likely more interested in LAI-PrEP when it becomes available. Continuing to build the evidence base will be crucial to understanding how to best deploy this new HIV prevention tool. As the HIV epidemic continues to evolve, communities continue to respond to it, and as more countries make LAI-PrEP available, ongoing research will be needed to understand the HIV prevention gaps that remain.

## Supplementary Material
